# Addressing Structural and Personal Barriers to Enhance Diversity in STEM Fields

**DOI:** 10.1093/geroni/igaf122.3260

**Published:** 2025-12-31

**Authors:** Maeva Laflamme, Melanie Horn Mallers, Jennifer Piazza, Laura Zettel-Watson

**Affiliations:** California State University, Fullerton, Irvine, California, United States; California State University, Fullerton, Fullerton, California, United States; California State University, Fullerton, Fullerton, California, United States; California State University, Fullerton, Fullerton, California, United States

## Abstract

As the U.S. population rapidly ages, the need for a diverse and highly trained MSTEM (Medicine, Science, Technology, Engineering, and Mathematics) workforce with expertise in aging science has never been greater. By 2030, adults over 65 will comprise more than 21% of the population, requiring innovative solutions in healthcare, biotechnology, and engineering (Colby & Ortman, 2014; Vespa et al., 2020). However, despite the increasing diversity of the population, MSTEM fields continue to lack representation from underrepresented minority students, first-generation students, and those from disadvantaged backgrounds (National Science Foundation, 2019). Much of the research on STEM disparities focuses on structural barriers, including financial constraints, access to advanced coursework, and systemic bias in academic institutions. However, personal barriers such as self-efficacy, sense of belonging, and psychological well being are often overlooked, despite their significant role in persistence and success (Matthews et al., 2020). Without a sense of belonging or personal confidence in their abilities, underrepresented MSTEM students may internalize failure, contributing to the “leaky pipeline” in STEM education. This study employs semi-structured interviews with 12 students participating in a two-year program focused on aging research. Students were asked seven open-ended questions about their experiences. The questions explored belonging/identity, and self-efficacy. Interviews were recorded for transcription and coding purposes. Results indicate that the two-year program significantly enhanced students’ sense of belonging by fostering a supportive community of individuals with shared goals and aspirations. Mentorship contributed to increased self-efficacy, and students reported feeling supported, attributing the program to improvements in their well-being.

